# 5-Aza-4’-thio-2’-deoxycytidine induces C&gt;G transversions in a specific trinucleotide context and leads to acute lymphoid leukemia

**DOI:** 10.21203/rs.3.rs-3186246/v1

**Published:** 2023-12-19

**Authors:** Peter Aplan, Ryan Bertoli, Yang Jo Chung, Michael Difilippantonio, Anthony Wokasch, Madison Marasco, Haley Klimaszewski, Susannah Garber, Yuelin Zhu, Robert Walker, Dengchao Cao, James Doroshow, Paul Meltzer

## Abstract

DNA methyltransferase inhibitors (DNMTi), most commonly cytidine analogs, are compounds that are used clinically to decrease 5’-cytosine methylation, with the aim of re-expression of tumor suppressor genes. We used a murine pre-clinical model of myelodysplastic syndrome based on transplantation of cells expressing a NUP98::HOXD13 transgene to investigate 5-Aza-4’-thio-2’-deoxycytidine (Aza TdCyd or ATC), a thiol substituted DNMTi, as a potential therapy. We found that ATC treatment led to lymphoid leukemia in wild-type recipient cells; further study revealed that healthy mice treated with ATC also developed lymphoid leukemia. Whole exome sequencing revealed thousands of acquired mutations, almost all of which were C > G transversions in a previously unrecognized, specific 5’-NCG-3’ context. These mutations involved dozens of genes well-known to be involved in human lymphoid leukemia, such as *Notch1, Pten, Pax5, Trp53* , and *Nf1* . Treatment of human cells *in vitro* showed thousands of acquired C > G transversions in a similar context. Deletion of *Dck* , the rate-limiting enzyme for the cytidine salvage pathway, eliminated C > G transversions. Taken together, these findings demonstrate that DNMTi can be potent mutagens in human and mouse cells, both *in vitro* and *in vivo* .

## Introduction

Myelodysplastic syndromes (MDS) are a diverse group of blood cancers characterized by peripheral blood cytopenias and dysplastic blood cell morphology [1,2]. Up to 40% of patients with high risk MDS will transform to acute myeloid leukemia (AML)[3], an aggressive blood cancer with significant morbidity and an overall 5-year survival of less than 35% [4]. MDS, AML, and blood cancers in general are thought to be driven by acquired and inherited mutations [5–7]. Acquired mutations in MDS, AML, as well as T cell and B cell Acute lymphoid leukemia (ALL) have recently been documented through next generation sequencing studies [8–13]. Both MDS and AML have been linked to hypermethylation of cytosine residues, a crucial process in normal biology [14]. Hypermethylation in the 5' regulatory region can result in epigenetic downregulation of gene expression, and it has been speculated that hypermethylation of tumor suppressor genes can contribute to malignant transformation [15].

DNA Methyl-Transferase Inhibitors (DNMTi), also referred to as hypomethylating agents (HMA), represent a class of drugs that inhibit DNA methylation through inactivation of DNMT1 [16]. Treatment with DNMTi is hypothesized to restore function to tumor suppressor genes that have become inactivated, slowing the progression of malignancy, often via induction of apoptosis [15]. One class of DNMTi are cytidine analogs, designed to mimic cytidine and inhibit the action of DNMT, most commonly DNMT1[16]. Two drugs of this class, 5-Azacytidine (5-AZA) and Decitabine (DAC), both of which are US Food and Drug Administration (FDA) have been approved for use in MDS [16]. However, the morbidity and mortality for patients with MDS or AML remain high, and thus new drugs are necessary to address this clinical need.

5-Aza-4'-thio-2'-deoxycytidine (Aza-TdCyd or ATC) is a cytidine analog that incorporates an aza modification (defined as a nitrogen in place of a carbon) of the cytosine base and a thioether modification of the deoxyribose sugar. ATC is a promising new DNMT1i that has demonstrated efficacy in preclinical studies of solid tumors [17–19]. Given this demonstration of efficacy in a pre-clinical model, and the use of similar cytidine analogs in MDS, we sought to characterize the potential utility of ATC in the treatment of MDS, using a murine NUP98::HOXD13 chimeric transplantation model.

## Results

**ATC Treatment of WT/NHD13 chimeric mice leads to T-ALL in both donor and recipient tissue.** To assess the *in vivo* pre-clinical efficacy of ATC, a newly described DNMT1 inhibitor, we generated a cohort of chimeric mice with both wild-type (WT) and MDS hematopoiesis. The chimeric mice were generated by co-transplantation of 200,000 WT and 1,000,000 NHD13 (MDS) bone marrow nucleated cells (BMNC) into WT recipients, following a myeloablative conditioning regimen of 900 cGy ionizing radiation. The NHD13 BMNC expressed the CD45.2 allele, while the WT BMNC and WT recipients expressed the CD45.1 allele, allowing us to distinguish hematopoiesis generated from WT and NHD13 cells using flow cytometry. Chimeric mice successfully engrafted both WT and NHD13 cells, as shown in Extended Data Fig. 1A-B; in addition, chimeric mice developed peripheral blood abnormalities consistent with MDS, such as macrocytic anemia and leukopenia (Extended Data Fig. 1C-D). Similar to the findings with NHD13 transgenic mice, 71% percent of NHD13/WT chimeric mice progressed to myelodysplastic syndrome or acute leukemia of donor origin, most commonly acute myeloid leukemia (AML; Ext Data Fig. 1E-G).

NHD13/WT chimeric mice were treated with either vehicle control (phosphate buffered saline; PBS) or ATC (1.0 mg/kg) once daily via intraperitoneal injection for up to 14 cycles of therapy, each cycle being two weeks of treatment followed by one week rest. Pooled results from three independent experiments revealed that ATC treated mice did not show prolonged survival, but instead showed reduced survival compared to PBS treated controls (Fig. 1A). Surprisingly, further analysis showed that over half of the ATC treated mice had developed leukemia of WT, not donor origin (Fig. 1B). Notably, leukemias that developed in WT cells were exclusively lymphoid, as opposed to myeloid leukemia that was seen in most NHD13 transformed cells. The most common form of lymphoid leukemia we identified was T cell acute lymphoblastic leukemia (T- ALL), also referred to as precursor T-lymphoblastic leukemia/lymphoma (pre-T LBL)[20] (Extended Data Table 1). The T-ALL cases typically showed thymic enlargement (Extended Data Table 1), along with peripheral blood abnormalities such as anemia, thrombocytopenia, and leukocytosis (Fig. 1C). More detailed analysis revealed invasion of lymphoblasts in the BM and spleen (Fig. 1D), and clonal T cell receptor beta (*Trb*) gene rearrangements (Ext Data Table 2; Fig. 1E). In sum, 72.7% of mice treated with ATC developed recipient T-ALL. Ionizing radiation, which was used as a preparative regimen, is well-established to be leukemogenic in mice; however, the 72.7% incidence is significantly higher than the 5–10% incidence of T-ALL seen in historical controls from our lab ($p = 0.0001$) (Fig. 1F).

**WES of ATC treated mouse leukemia reveals marked increase in C > G transversion.** We utilized whole exome sequencing (WES) to search for acquired mutations in the T-ALL samples. Remarkably, we found a dramatic increase in both number (mean +/- standard deviation of 762 +/- 642 vs 2 +/- 1; Mann-Whitney $p = 0.003189$) and percentage (89 +/- 5 vs 7.5 +/- 2.5; Mann-Whitney $p = .003318$) of C > G transversions in all ATC treated samples, with up to thousands of acquired mutations per sample (Fig. 2A-B, Supp Data Table 1). Further characterization of the acquired mutations using Single Base Substitution (SBS) profile software (SigProfiler MatrixGenerator, Extractor, Simulator, and Plotter) demonstrated that the C > G transversions occurred almost exclusively in a 5'-NCG-3'context (Fig. 2C). This signature was not recognized as an existing signature present in the COSMIC database by the SigProfiler software, which led us to regard this as a novel SBS signature[21–25]; we have provisionally designated this new signature as SBS-ATC. Figure 2C shows the stark difference between T-ALL in a PBS treated mouse, with 1 C > G transversion, as opposed to T-ALL in an ATC treated mouse, with > 1000 C > G transversions. Additional evidence for the specificity of ATC associated mutations comes from analysis of two mice that developed early thymocyte precursor (ETP) ALL in donor NHD13 cells (Fig. 2D; Extended Data Fig. 2). Close examination of these ETP samples demonstrated that these two leukemias had identical, clonal D-J rearrangements, indicating that a pre-malignant ETP clone had been transplanted to the recipient mice; this model is consistent with a recent report that NHD13/$IDH2^{R140Q}$ mice frequently develop ETP ALL [26]. However, despite the fact that these two donor ETP originated from the same pre-malignant clone, the ETP-ALL clone in a PBS treated mouse acquired only two C > G transversions, whereas an ETP clone from an ATC treated mouse demonstrated over 2000 acquired C > G transversions (Fig. 2D). The reproducibility of the SBS-ATC signature is evident in Supp Fig. 1A, in which a marked increase in C > G transversions, in a 5'-NCG-3' context, is seen in every leukemia from mice treated with > 0.1 mg/kg of ATC.

We employed PCR amplification and Sanger sequencing to verify a subset of the acquired C > G transversions that were identified by NGS; in all cases tested, the C > G transversions were detected by Sanger sequencing (Ext data Fig. 3A). In a smaller subset, we verified that the C > G transversions were indeed transcribed into mRNA (Ext data Fig. 3B). The detection of these mutations in both gDNA and cDNA using Sanger sequencing demonstrated that these highly specific C > G transversions were *bona fide* mutations and not sequencing artefacts.

**ATC treatment of transplanted mice leads to recipient (r) T-ALL in the absence of ionizing radiation.** Given the use of myeloablative ionizing radiation (IR) in generating the NHD13 chimeric model, and the known mutagenic potential of IR, we sought to examine a requirement for IR in the unique C > G transversions characterized above. To avoid IR, we used CASIN as a non-genotoxic conditioning regimen [27]. CASIN, for Cdc42 activity-specific inhibitor, treatment leads to egress of WT hematopoietic stem and progenitor cells (HSPC) from the recipient BM, allowing for engraftment of transplanted HSPC [28]. We also varied the dosage of ATC in this experiment to investigate the possibility of a dose-dependent effect on C > G transversion. CASIN conditioning led to successful engraftment of NHD13 HSPC (Fig. 3A, Extended Data Table 2).

The engraftment of NHD13 HSPC varied with ATC dosage, as higher ATC doses were associated with a lower median engraftment; this may have been due to effective treatment of the Cd45.2 + NHD13 MDS (Fig. 3A). Similar to the transplants using IR, we noted a highly penetrant phenotype; all mice treated with either 0.5 mg/kg or 1.0 mg/kg ATC developed rT-ALL (Fig. 3B). In contrast, only one of six mice treated with 0.1 mg/kg ATC developed rT-ALL, and none of six PBS control mice developed rT-ALL. All mice treated with PBS developed donor MDS/AML, confirming the high penetrance of MDS/AML in recipients of NHD13 BMNC (Fig. 3B, Extended Data Tables 1 and 2). Two ATC-treated mice developed a concurrent donor AML and a recipient T – lineage ALL; this is not surprising given the highly penetrant nature of both ATC treatment and NHD13 BMNC transplantation (Supp Fig S2).

Similar to the results with transplants that employed IR, WES of leukemia from ATC treated mice following CASIN conditioning showed hundreds to thousands of C > G transversions (Fig. 3C, Supp Data Table 2) and that almost all SNV were C > G transversions (Fig. 3D). In addition, the number of C > G transversions increased with increasing ATC dosage (Fig. 3C). The unique 5’-NCG-3’ context for the C > G transversion was reproduced in all ATC treated mice that received CASIN conditioning (Fig. 3E; Supp Fig. 1B). These results demonstrate that the C > G transversion and induction of lymphoid leukemias in ATC treated NHD13 chimeric mice was not dependent on IR.

## ATC induces C > G transversions and T-ALL in non-transplanted WT mice

There is evidence that both human [29,30] and murine [31] MDS hematopoietic stem and progenitor cells (HSPC) cells can “re-shape” the wild-type BM microenvironment. To eliminate the possibility that a BM microenvironment re-shaped by NHD13 HSPC was required for ATC-induced mutagenesis, we treated non-transplanted WT C57BL6 mice with ATC. Given that the thymus typically involutes with age, we also wished to determine if age effected the susceptibility to ATC-induced T-cell leukemogenesis. Three independent experiments were conducted using WT mice aged 2, 8, or 12–15 months and 1.0 mg/kg ATC. Sublethal IR (600 cGy) was also included in some experiments to allow for comparison to prior experiments with ATC and IR.

All three age groups showed significantly decreased survival (Figs. 4A-C) for both ATC alone and ATC + IR treatment, with ATC + IR consistently showing poorer survival. A pooled survival curve along with cause of death is shown in Fig. 4D. All mice that received ATC, with or without IR, were either found dead or developed lymphoid leukemia within 27 weeks of beginning ATC treatment, with the majority of mice developing lymphoid leukemia between 15–20 weeks. This peak corresponds to 5–6 cycles of ATC treatment.

Recipient T-ALL in non-transplanted WT mice treated with ATC was similar to that which developed in the NHD13 transplant recipients, with invasion of T-lymphoblasts in BM and spleen (Ext Data Fig. 4A), as well as non-hematopoietic tissues such as kidney and liver (Ext Data Fig. 4B), and clonal *Trb* gene rearrangements (Ext Data 4C-D). In addition to T-ALL, we also observed B-ALL in a smaller number of mice, suggesting the possibility that ATC treatment could be oncogenic in B as well as T lymphoblasts. The B-ALL were characterized by anemia, thrombocytopenia, and leukocytosis. (Supp Fig S3A). Flow cytometry showed invasion of $CD19^{+}/B220^{+}$ lymphoblasts in the BM, spleen, and lymph nodes (Supp Fig S3B) as well as non-hematopoietic tissue such as liver and kidney (Supp Fig. 3C). Further supporting a diagnosis of B-ALL was a clonal *Igh* gene rearrangement (Supp Fig. 3D), and establishment of immortal

B-ALL cell lines from some of these samples. A small subset of ATC-treated WT mice developed concurrent T- and B-ALL. These mice again showed anemia, thrombocytopenia, and leukocytosis (Supp Fig S3E), and invasion of clonal B cells in BM and spleen and T cells in the thymus (Supp Fig S3F-G). These were two independent leukemias, as there were only nine mutations shared between the T-ALL and B-ALL, as compared to 6272 mutations that were not shared (Supp Fig. 3H). Acquired mutations included those associated with murine T-ALL (*Notch1*) in the thymus and murine B-ALL (*Bcor*) in the BM (Supp Fig. 3I).

WES (Supp Data Table 3) of leukemias that arose in ATC treated mice revealed increased number and percentage of C > G transversions (Fig. 4E-F) similar to prior experiments, once more in a 5'-NCG-3' context (Supp Fig. 1C). These results demonstrate that neither IR nor NHD13 transplantation was required for the induction of lymphoid leukemia by ATC; and that ATC treatment could induce B-ALL as well as T-ALL in WT mice (Fig. 4D).

**WGS shows correlation of ATC-induced C > G transversions and global CpG density.** We used Whole Genome Sequencing (WGS) (Supp Data Table 4) to map the location of C > G transversions on a subset of eight ATC-induced T-ALL samples. We detected an average of more than 24,000 mutations per sample, primarily C > G transversions in a 5'-NCG-3' context. As shown in Supp Fig. 1D, despite a considerable difference in the total number of SNV (range 3,865 – 42,867), the mutation profiles of these samples are almost identical. Using the ChromoMap package in R, we mapped pooled C > G transversion density as well as each individual mouse C > G transversion density [32,33] (Supp Fig. 4A-H). Given the 5'-NCG-3' context of C > G transversions, we hypothesized that C > G transversion would correspond to known CpG density. We found that while C > G transversion dense areas generally mapped to CpG dense regions, the highest density of C > G transversions did not invariably map to the highest density of CpG dinucleotides (e.g., the distal portion of chromosome 2 in Fig. 5). In addition, not every CpG dense region had high C > G transversion density (e.g., chromosome 18 in Fig. 5), and there is considerable heterogeneity in C > G transversion mapping when examining individual mouse samples (Supp Fig S4A-H). We also found increased C > G transversion density in genomic regions that contain known cancer-associated genes, such as *Notch1, Ikzf1*, and *Trp53* (Fig. 5), suggesting that C > G transversion may lead to *in vivo* selection due to mutations that confer a fitness advantage. Nonetheless, Supp Data Table 5 reveals that among 193,839 total mutations detected by WGS, only 281 (0.145%) C > G transversions occurred in the exact same nucleotide position among two different mice, and no exact nucleotide position was mutated more than twice; therefore, it seems ATC does not preferentially mutate at any single nucleotide position, but rather acts preferentially within larger chromosomal regions.

**Numerous C > G transversions occur in genes relevant for human lymphoid leukemia.** To investigate a relationship between C > G mutagenesis and lymphoid oncogenesis, we determined whether C > G transversions commonly occurred in genes associated with human cancer, especially lymphoid malignancy. To avoid complexities introduced by IR and NHD13 transplant, we focused these studies on 22 lymphoid leukemias (19 T-ALL and 3 B-ALL) that were generated by ATC treatment of WT mice. We compared Tier 1 mutations identified in this set to a set of 432 genes commonly associated with cancer that were part of the FoundationOne® Heme Gene panel (Foundation Medicine, Inc.) used to detect relevant cancer mutations in human hematologic malignancy. This analysis identified a total of 612 Tier 1 C > G transversions in genes associated with cancer in the 22 mouse samples for an average of 28 potentially oncogenic mutations per leukemia sample (Fig. 6A, Supp Data Table 6). Additionally, the 40 genes most commonly mutated included genes well known to be associated with human lymphoid malignancy, such as *Bcl11b*, *Ikzf1*, *Trp53, Pten*, *Kras, Jak3*, and *Notch1* (Fig. 6A). Further analysis of C > G transversion position revealed that amino acid mutations often occurred in known oncogenic "hotspots", such as *Trp53* R270P, homologous to human R273 mutants (Fig. 6B), *Pax5* P80R (Fig. 6C), and *Pten* R130G (Fig. 6D)[34] These results indicate that the induction of C > G transversions in lymphoid cells is the likely proximal cause of leukemic transformation observed in ATC treated mice.

**C > G transversions can be generated in human cells after brief ATC exposure in vitro**. We treated the human AML cell line U937 with ATC *in vitro* (Fig. 7A) to address two questions; 1) were human cells susceptible to the mutagenic effect of ATC, and 2) could we develop an *in vitro* assay for ATC-induced mutagenesis. To minimize diversity of the initial U937 cell population, we first single cell cloned the U937 cell line. The cloned U937 parental line was then treated for 6 days in vitro with 25, 50, or 100nM ATC or PBS. Reasoning that DNA harvested at this time may contain multiple populations of mutagenized U937 cells, we then single cell cloned the treated cells, and harvested genomic DNA from both the bulk U937 population as well as the individual single cell clones.

WES (filtered for VAF > = 0.2 and alternate allele count > = 5) from bulk ATC treated cells revealed 0 SNV. However, there was a dramatic difference in number and percent of C > G SNV in the ATC treated individual clones. The ATC treated clones had 495 ± 360 C > G transversions per clone (Fig. 7B), whereas the PBS treated clones had 0.2 ± 0.4 C > G transversions per clone, p = 0.009701, a difference of > 1000-fold (Supp Data Table 7). There were similar differences in the percent C > G SNV among all mutations (Fig. 7C). SBS analysis revealed a similar 5'-NCG-3' mutational context in human cells, but without the 5'-TCG-3' peak as observed in mice (Fig. 7D, Supp Fig. 5). We refer to this in vitro assay as GEMINI for Genotoxic Mutation Signature Identified After Clonal Expansion In Vitro.

The human T-cell line CEM was also examined using the GEMINI assay. Similar to U937 cells, we noted a marked increase in C > G transversions in ATC treated single cell clones (1172 ± 447 vs 55 ± 38, p = 0.007937) (Ext Data Fig. 5A). However, in contrast to treatment of U937 cells, in which the percent of C > G transversions was 86–91% (Fig. 7C), the percent of mutations that were C > G transversions in ATC treated CEM clones was much lower, only 26–45% (Ext Data Fig. 5B). Moreover, the total number of variants in the CEM PBS control single cell clones was far higher than the U937 PBS single cell clones (1215 ± 194 vs. 0.25 ± 0.4, p = 0.01193)(Supp Data Table 7). However, it has previously been reported that the CEM cell line has a mismatch repair deficiency due to deletion of *MLH1*[35]. Examination of the CEM WES .bam files revealed an almost total absence of *MLH1* reads, consistent with a homozygous *MLH1* deletion. SBS analysis of the CEM clones identified two signatures associated with mismatch repair deficiency (COSMIC signatures SBS15 and SBS21), as well as a novel, previously unreported signature, similar to that seen in U937 cells. Inspection of the SBS profiles for CEM reveals C > G and C > T SNVs, both preferentially in a 5’-VCG-3’ context (V indicating not T) (Ext Data Fig. 5C) (See Supp Fig. 5 for all SBS plots). Taken together, these results demonstrate that ATC can induce C > G transversions in human cells.

In addition to ATC, we evaluated the DNMTi decitabine (DAC; FDA approved for treatment of MDS) for a potential mutagenic effect, as both molecules are deoxycytidine analogs with Aza moieties in the cytosine base. WES using the protocol outlined in Fig. 7A revealed an increase in the number (10 ± 6 vs. 0.2 ± 0.4, p = 0.009701) (Ext Data Fig. 6A) and proportion (20–70%, p = 0.009701)(Ext Data Fig. 6B) of C > G mutations in DAC treated vs PBS controls (Supp Data Table 7). However, this effect was markedly reduced compared to ATC, and no clear 5’-NCG-3’ or 5’-VCG-3’ signature was observed in DAC treated samples (Ext Data Fig. 6C) (See Supp Fig. 5 for all SBS plots). These results suggest that DAC may have a similar, but weaker mutagenic effect compared to ATC.

**Dck is required for C > G transversions induced by ATC.** It is unclear why ATC induced only lymphoid malignancy in the *in vivo* studies. Given that ATC is an unphosphorylated cytidine analog, we reasoned that ATC would need to be phosphorylated to be incorporated into DNA. Phosphorylation of deoxycytidine is mediated by deoxycytidine kinase (*Dck)*, the rate-limiting enzyme in the cytosine “salvage” pathway (Fig. 8A) [36]. Dck is most highly expressed in lymphoid tissue [37,38] and its importance in lymphoid cell development is underscored by the observation that the only phenotype noted in *Dck* KO mice was in T and B cell precursors [39]. We thus hypothesized the expression of *Dck* in lymphoid tissue allowed for incorporation of ATC into the lymphoid cell genome, leading to C > G mutations and leukemogenesis.

To assess whether phosphorylation of ATC by Dck was required for the mutagenic effect of ATC, we utilized a murine T-ALL cell line with a homozygous deletion of *Dck* that had been generated by serial passage in the presence of cytarabine; the parental cell line is designated 7298, while the cytarabine resistant cell line is designated 7298CR (Fig. 8B-C). A dose-dependent effect on both cell growth (Fig. 8D) and viability (Fig. 8E) was evident in the parental 7298 (*Dck* WT) cell line, whereas the 7298CR (*Dck* deleted) cell line showed little effect at any concentration of ATC tested. We used the GEMINI assay described in Fig. 7A to generate single cell clones of both the 7298 and 7298CR cell line following treatment with 1000 nM ATC. WES revealed a marked increase in both the number (Fig. 8F) and proportion (Fig. 8G) of C > G transversions in the 7298 clones, whereas 7298CR clones had very few C > G transversions (Supp Data Table 8). SBS profiles of the 7298 clones demonstrated the same 5’-NCG-3’ context that was identified in the murine T- and B-ALL samples, whereas profiles of the 7298CR clones had very few C > G transversions (Fig. 8H) (Supp Fig. 6). These results demonstrate that Dck expression is required for ATC induced C > G mutagenesis and suggest that lymphoid leukemia induction in the context of ATC treatment may be due to high Dck expression in lymphoid cells.

## Discussion

The results of this study reveal a highly penetrant mutagenic and carcinogenic phenotype associated with ATC, an investigational DNMTi. The observation of lymphoid leukemias with or without either

NHD13 co-transplantation or ionizing radiation demonstrates that ATC exposure is sufficient to induce lymphoid leukemia. Furthermore, sequencing of these leukemias revealed C > G transversions in a unique 5'-NCG-3' context. The finding of C > G transversions encoding missense and nonsense mutations within well-known cancer genes, often at residues that are recurrently associated with human lymphoid leukemia, provides compelling evidence that the mutagenic phenotype is directly linked to malignant transformation of murine lymphoblasts.

The discovery of a previously unrecognized mutational signature associated with ATC treatment highlights the complexity and non-random nature of SBS mutational processes. Other mutational signatures induced by chemotherapy treatment have been characterized, including temozolomide (SBS11)[21,40], and cisplatin (SBS34, SBS35)[41]; in addition, the toxin aristolochic acid generates a highly specific signature (SBS22)[24,42,43]. However, none of these agents are characterized by a striking C > G transversion preference.

The unique C > G transversion following ATC treatment offers the potential to study this form of mutation in the context of malignant transformation. Among the six potential types of SBS, the most common form of SBS found in human cancer is a C > T transition, likely caused by spontaneous deamination of a 5'-methylcytosine (designated SBS1)[23]. A C > T transition can produce certain amino acid substitutions based on the mammalian genetic code, whereas other amino acid substitutions cannot be produced by a C > T transition; for example, a C > T transition cannot produce a Pro > Arg substitution, whereas a C > G transversion can produce a Pro > Arg substitution. This fact makes it more difficult to study cancers which are driven by less common missense or nonsense mutations that can not be generated by a C > T transition. The ability to induce thousands of C > G transversions makes ATC a potential tool for the detection and validation of less common oncogenic amino acid substitutions that are underrepresented in current databases, which in turn offers promise in understanding oncogenic protein variants.

The leukemias that developed in WT (including recipient cells for transplant experiments) cells were exclusively of T or B lymphoblast origin. However, despite the absence of WT AML arising in ATC treated cells, AML did occur in NHD13 donor cells of transplant recipients treated with ATC, and these AML samples showed the same unique C > G transversions in a 5'-NCG-3' context, indicating an ATC mutagenic effect in myeloid as well as lymphoid cells. In addition, the *in vitro* experiments with U937 (myeloid) cells demonstrates that ATC can be mutagenic in myeloid cells as well as lymphoid cells. We speculate that the predilection for lymphoid leukemias may be due to elevated expression of Dck in lymphoid tissues, potentially due to higher usage of the lymphoid salvage pathway in lymphoid precursor cells [39].

The use of single cell colonies to isolate and amplify a unique mutational profile which is undetectable by WES of bulk populations of cells treated *in vitro* demonstrates the power of single cell clones for the detection and study of mutagenic compounds. Current techniques commonly used to evaluate mutagenicity *in vitro* include the Ames test[44], the HPRT assay [45], and the TK assay [46], and assessment of BigBlue™ [47] mice *in vivo*. While these tests are capable of detecting SBS and deletions within select genes, none are able to characterize the prevalence of mutations across the genome or a specific mutational signature associated with a mutagenic agent in question. Thus, we speculate that the *in vitro* GEMINI assay described in this manuscript could be useful in future studies of mutational processes associated with chemical or biological agents in human cells.

Although not the focus of this manuscript, the *in vitro* assay demonstrated a significant 40-fold increase in C > G transversions in U937 cells treated with decitabine vs PBS treatment. This finding is consistent with the observation that some MDS patients show increased C > G transversions following treatment with decitabine or 5-azacytidine [48,49]. Moreover, a small number of patients with either AML [50] or solid tumors [17,50] have responded to treatment with immune checkpoint inhibitor therapy either with or following 5-azacytidine treatment [50,17]. The interpretation of these observations has been that reactivation of endogenous antigens following 5-azacytidine treatment leads to additional antigenic targets and resultant sensitivity to immune checkpoint inhibitors [51]. The data presented in this manuscript suggests a potential alternate mechanism, namely, that increased antigenic targets caused by multiple C > G transversions results in increased sensitivity to immune checkpoint inhibitors.

In sum, this study demonstrates that ATC is a potent mutagen capable of inducing C > G transversions, in a unique 5'-NCG-3' context, in murine cells or a unique 5'-VCG-3' context in human cells. The collection of C > G transversions is capable of inducing B and T-ALL in mice, regardless of transplant or IR exposure. These mutations arise predominantly within CpG islands, are reproducible in multiple studies, and are confirmed by orthogonal assays such as Sanger sequencing of DNA and RNA. We propose that ATC can be a useful reagent for future studies in characterizing C > G transversions and cancers arising from these transversions and highlight the potential utility of *in vitro* assays of clonal expansions for identifying mutagenic potential in mammalian cells.

## Online Methods

**Mouse strains, genotyping, and identification.** NUP98::HOXD13 (NHD13) transgenic donor mice were generated on a C57BL/6 Cd45.2 background and genotyped as previously reported [39]. Chimeric mice with both wild-type and MDS-derived hematopoiesis were generated using a bone marrow transplantation model as previously reported [52]. Recipient mice were purchased from Jackson Laboratory. Mice were labelled with ear tags, and ear punches were employed if tags were removed by the animals during the experiment. All animal experiments were approved by the National Cancer Institute (Bethesda) Intramural Animal Care and Use Committee.

**Drug treatment and schedule.** 5-Aza-4'-thio-2'-deoxycytidine (Aza-TdCyd or ATC, NSC 777586 – CAS 169514-76-5) was obtained from the Drug Synthesis & Chemistry Branch, Developmental Therapeutics Program, Division of Cancer Treatment and Diagnosis, National Cancer Institute. Stock solutions of 1.169 uM were prepared and stored as aliquots at -20 Celsius. Aliquots were thawed and used once and not refrozen. 5-aza-2'-deoxycytidine (Decitabine or DAC) was obtained from Millipore Sigma (Cat#1165204), and stock solutions of 0.468 uM were prepared and stored as aliquots at -20 Celsius. Aliquots were thawed and used once and not refrozen. The ATC *in vivo* treatment schedules utilized a 3-week treatment cycle. Following disinfection of the anterior abdominal wall with topical ethanol, mice received an intraperitoneal injection of ATC or vehicle (phosphate buffered saline; PBS) control. Mice were held in a supine position during injection as to minimize risk of organ puncture. Injections would be repeated for the next four days (a total of 5), followed by two days' rest. Injections were then given for the next 5 days, followed by 9 days of rest. This treatment constituted one cycle of treatment. Treatment continued until the mice succumbed to leukemia or MDS, for as many as 14 cycles.

**Leukemia assessment.** Peripheral blood from tail veins was collected before each treatment cycle. Complete Blood Counts (CBCs) were analyzed with a HEMAVET Multispecies Hematology Analyzer (CDC Technologies). Mice that exhibited clinical symptoms consistent with severe MDS or AML, such as hunched posture, lethargy, cachexia, tachypnea, or abscess formation were euthanized via CO2 asphyxiation. Following euthanasia or natural death, mice would be examined for signs of disease, including organomegaly, tumor masses, and pleural effusion. Organs such as spleen and thymus were collected and suspended in Hank's balanced salt solution supplemented with 2% fetal bovine serum (HF2) for further immunophenotypic analysis. Bone marrow was flushed from the long bones using HF2, and indicated organs intended for histological analysis were immediately placed in 10% formalin solution. Diagnoses of hematologic malignancies was conducted according to published guidelines for murine leukemia [53].

**Flow cytometry.** Flow cytometry was performed as described previously [52]. Single cell suspensions were prepared from each tissue: bone marrow was flushed from long bones as described above, and spleen and thymus tissue was gently teased apart and filtered through a 40-uM mesh filter into HF2. Cells were counted and resuspended in an HF2 solution containing 5% rat serum solution to block binding to Fc receptors. Cells were then incubated with a cocktail of antibodies for 30 minutes at 4 degrees Celsius. The antibody cocktail included: Mac-1/PE (clone-M1/70), BioLegend, CD71/PE/Dazzle594 (clone-R172170), BioLegend, CD45.2/Alexa Fluor 532 (clone-104), Invitrogen, B220/PerCP-eFluor 710 (clone-RA3-6B2), Invitrogen, Sca-1/PE-Cy7 (clone-D7), eBioscience, Ter119/APC (clone-TER119), BioLegend, CD19/Alexa Fluor 700 (clone-6D5), BioLegend, Viability, 7AAD, BioLegend, Kit/APC-eFluor 780 (clone-2B8), Thermo Fisher, CD4/PerCP-Cy5.5 (clone-GK1.5), BioLegend, and CD8/APC-Cy5.5 (clone-53-6.7), Abcam. After incubation, cells were washed in PBS and resuspended in HF2. Cells were then analyzed using a Cytek Northern Lights cytometer.

**Histology and immunohistochemistry (IHC).** Organs and tissues were placed in 10% formalin and embedded in paraffin. Paraffin sections were stained with hematoxylin and eosin (H&E), myeloperoxidase (Cat#A0398; Dako), CD3 (Clone-CD3-12, Bio-Rad), CD45R/B220 (Clone-RA3-6B2, BD Biosciences), and Ter119 (Clone-TER119, BioLegend). Sections were scanned using an Aperio At2 digital slider scanner (Leica Biosystems) and stored in a database within the eSlide Image Management System. Scans were viewed using Aperio ImageScope v12.4.6.5003 software.

**Cell culture.** Human U937 and CEM cell lines were obtained from American Type Culture Collection (ATCC), cultured as single cell suspensions in Iscoves modified Dulbecco Media (IMDM) media supplemented with 10% fetal bovine serum (FBS), glutamine, and penicillin/streptomycin (hereafter referred to as IMDM complete media), and maintained as previously reported [54,55]. The 7298 cell line is a murine T-ALL cell line derived from SCL/LMO1 transgenic mice [54] and was grown in IMDM complete media. The 7298CR cell line is a cytarabine resistant derivative of 7298 that has a biallelic deletion of Dck (D. Cao and P.D. Aplan, unpublished). All cells were maintained at 37 C and 5% CO2.

**In vitro drug treatment (GEMINI assay).** Cell lines were expanded and treated in 25 mL flasks at a concentration of 50,000 cells/mL in 5mL of IMDM complete media. Cells were treated for 3 days with drug, then washed, resuspended in fresh media, and treated for an additional 3 days. Following a 4-day rest period, single cell clones were expanded in 96 well plates as follows. Treated cells were collected by centrifugation and resuspended at a concentration of 5 cells/mL. 100 uL (or an average of 0.5 cells/well) were then plated in 96 well plates and expanded for one week. In a typical experiment, 40–50 wells would expand; 5–10 clones were then picked at random to expand for whole exome sequencing (WES). Cell counts and viability were conducted using an automated cell counter (TC20 Automated Cell Counter, BioRad) and Trypan Blue Viability assay (Lonza BioWhittaker Trypan Blue 0.4%, Fisher Scientific, Cat# BW17-942E).

**Nucleic acid extraction, reverse transcription, polymerase chain reaction (PCR), and Sanger sequencing.** Genomic DNA was extracted from each tissue using the DNeasy Blood & Tissue kit (Qiagen) and the manufacturer's suggested protocol. RNA was isolated using TRIzol Reagent and the accompanying RNA extraction protocol (Cat#15596026, Invitrogen). Reverse transcription of mRNA was accomplished using the SuperScript III First-Strand Synthesis System (Invitrogen, Cat#: 18080-051) and accompanying protocol. PCR amplification to identify clonal murine T cell receptor beta (*Trb*) rearrangements used mRNA or DNA templates and primers and protocols as previously described[26]. Clonal Igh gene rearrangements were identified using primers and protocols as previously described [56]. Gene specific amplification to verify mutations used DNA or mRNA templates and primers listed in Supp Data Table 9. Primers for cDNA PCR were designed to reside on differing exons as to avoid false positive signals from possible contaminating gDNA. Amplification of the murine *Scid* locus was used as a quality control for genomic DNA, and amplification of the murine *Actb* gene was used as a quality control for mRNA. All PCR amplification assays used LiTaq PCR mix (Cat# M0024, LifeSct) and the manufacturer's recommended protocol. Primers are listed in Supp Data Table 9. Amplicons were size fractionated on agarose gels and purified using the Qiagen QIAquick PCR Purification Kit (cat#28104). Sanger sequencing of purified fragments was performed by the NCI Sequencing Core. Clonal *Trb* and *Igh* rearrangements identified using PCR were confirmed by inspection of .bam files using an Integrated Genome Viewer (IGV, Broad Institute v 2.12.3) for samples in which WES results were available.

**Western Blot**. SCL/LMO1 parental 7298 cells and cytarabine resistant 7298 cells were lysed in RIPA lysis buffer (sc-24948A, Santa Cruz) with 1 mM sodium orthovanadate, 1 mM PMSF and protease inhibitor cocktail on ice for 15 min. The protein concentration of sample is determined by Micro BCA™ Protein

Assay Kit (Cat#23235, Thermo Fisher Scientific). The protein sample is prepared with Laemmli buffer (Cat#1610747, BIO-RAD) to a final concentration of 1 ug/ul. 20 µl protein samples were applied to 10% SDS-PAGE gels, separated by electrophoresis at 80 V for 30 min and 120 V for 90 min. After electrophoresis, protein was transferred to a nitrocellulose membrane using Semi-dry Transfer System (Power Blotter, Thermo Fisher Scientific). The membrane was then blocked in 5% non-fat dry milk (Blotting-Grade Blocker, BIO-RAD) in TBST (10 mM Tris–Cl, 150 mM NaCl, 0.05% Tween-20, pH 7.5) for 1 h. The membrane was then incubated with primary antibody overnight at 4°C followed by incubation with a secondary antibody conjugated to horseradish peroxidase (HRP), and visualized using a chemiluminescence kit (GE Healthcare, RPN2106). Specific antibodies to DCK (Cat# ab186128, Abcam), β-Actin (Cat# A1978, Sigma) were used to detect protein levels. β-Actin was used as a loading control.

**Whole-Exome Sequencing.** Genomic DNA (500 ng) was sheared to a mean size of 300 bp on a S2 Covaris Ultrasonicator. Illumina libraries were prepared using a KAPA HyperPrep Kit, according to the manufacturer's instructions, incorporating unique dual indexes (IDT). Mouse Illumina libraries were pooled and enriched using Agilent SureSelect XT System with Mouse All Exon baits according to the manufacturer's instructions. Human Illumina libraries were pooled and enriched using IDT xGen Human Exome Lockdown Probes and Reagents according to the manufacturer's instructions. Enriched libraries were sequenced on an Illumina NextSeq 2000 Sequencing System using P3 (300 cycle) reagents. Data processing and variant calling procedure followed the Best Practices workflow recommended by the Broad Institute. Briefly, the raw sequencing reads were mapped to mouse genome build 10 (mm10) by the Burrows-Wheeler Aligner [57] followed by local realignment using the GATK suite v4.2.5.0 9 [58] from the Broad Institute, and the Picard tools marked duplicated reads. The somatic variants were called by MuTect2 within the GATK suite. The somatic variants were first filtered with the GATK recommended filtering criteria and further filtered by the following criteria: baitRegion= "TRUE", FILTER = "PASS", TUMVAF > = 0.2, and AD_TUMOR > = 5. The filtered variants were used for the Mutational Signature analysis, for which the software SigProfilerMatrixGenerator v1.2 and SigProfilerExtractor v1.1.4 [59] from AlexandrovLab at UCSD were used.

**Whole-Genome Sequencing.** Whole genome sequencing was conducted at the NCI Frederick ATRF (Advanced Technology Research Facility). Analysis of mapped sequence reads (percent duplicate reads 12–31%, mean coverage of 62-123X) using Mutect 2 software and parameters was done as described above for WES. Generation of ChromoMaps was conducted using the R package ChromoMap v4.1.1 [32] using R Statistical Software version 4.3.0 [33].

**Statistics and Hypothesis Testing.** Data are reported as means ± standard deviation unless otherwise noted. Survival curves were generated using GraphPad Prism v8.4.3 (GraphPad Software, LLC) and were analyzed using the Mantel-Cox Log-Rank test for significance. The Bonferroni correction was used to correct for multiple hypothesis testing where applicable. Comparisons between mutation counts and percentages were conducted using the Mann-Whitney U test. P values of < 0.05 were considered statistically significant in the context of this study.

### Declarations

## Conflict of Interest:

PDA receives royalties from the NIH Technology Transfer program for the invention of NHD13 mice.

## Acknowledgments:

The authors thank current and former members of the Aplan lab, Andre Nussenzweig, and Michael Kuehl (deceased) for insightful discussions. We thank the NCI Sequencing Minicore for Sanger sequencing, the NCI Transgenic Core for generation of transgenic mice, the NCI Genomics Core for Next Generation Sequencing, the NCI Flow cytometry core for cell sorting, the NCI Pathology/Histotechnology Lab (PHL) for immunohistochemistry, Shelley Hoover and Mark Simpson of the NCI Molecular Pathology Unit for assistance with slide imaging, and Maria Jorge for excellent animal husbandry. This work was supported by the Intramural Research Program of the National Cancer Institute, National Institutes of Health (grant numbers ZIA SC 010378 and BC 010983) and the NCI Experimental Therapeutics (NExT) program.

## References

1. Cazzola, M. Myelodysplastic Syndromes. *N Engl J Med* **383**, 1358-1374 (2020).
2. Nimer, S.D. Myelodysplastic syndromes. *Blood* **111**, 4841-51 (2008).
3. Kota, V. *et al.* Clinical impact of transformation to acute myeloid leukemia in patients with higher-risk myelodysplastic syndromes. *Future Oncology* **18**, 4017-4029 (2022).
4. Kantarjian, H. *et al.* Acute myeloid leukemia: current progress and future directions. *Blood Cancer J* **11**, 41 (2021).
5. Sperling, A.S., Gibson, C.J. & Ebert, B.L. The genetics of myelodysplastic syndrome: from clonal haematopoiesis to secondary leukaemia. *Nat Rev Cancer* **17**, 5-19 (2017).
6. Iacobucci, I. & Mullighan, C.G. Genetic Basis of Acute Lymphoblastic Leukemia. *J Clin Oncol* **35**, 975-983 (2017).
7. De Kouchkovsky, I. & Abdul-Hay, M. 'Acute myeloid leukemia: a comprehensive review and 2016 update'. *Blood Cancer Journal* **6**, e441-e441 (2016).
8. Yu, J. *et al.* Gene mutational analysis by NGS and its clinical significance in patients with myelodysplastic syndrome and acute myeloid leukemia. *Experimental Hematology & Oncology* **9**, 2 (2020).
9. Ryan, S.L. *et al.* Whole genome sequencing provides comprehensive genetic testing in childhood B-cell acute lymphoblastic leukaemia. *Leukemia* **37**, 518-528 (2023).
10. Zhang, J. *et al.* The genetic basis of early T-cell precursor acute lymphoblastic leukaemia. *Nature* **481**, 157-163 (2012).

11. Brady, S.W. *et al.* The genomic landscape of pediatric acute lymphoblastic leukemia. *Nature Genetics* **54**, 1376-1389 (2022).
12. Papaemmanuil, E. *et al.* Genomic Classification and Prognosis in Acute Myeloid Leukemia. *New England Journal of Medicine* **374**, 2209-2221 (2016).
13. Haferlach, T. *et al.* Landscape of genetic lesions in 944 patients with myelodysplastic syndromes. *Leukemia* **28**, 241-247 (2014).
14. Moore, L.D., Le, T. & Fan, G. DNA methylation and its basic function. *Neuropsychopharmacology* **38**, 23-38 (2013).
15. Esteller, M. CpG island hypermethylation and tumor suppressor genes: a booming present, a brighter future. *Oncogene* **21**, 5427-40 (2002).
16. Mehdipour, P., Chen, R. & De Carvalho, D.D. The next generation of DNMT inhibitors. *Nat Cancer* **2**, 1000-1001 (2021).
17. Wrangle, J. *et al.* Alterations of immune response of Non-Small Cell Lung Cancer with Azacytidine. *Oncotarget* **4**, 2067-79 (2013).
18. Thottassery, J.V. *et al.* Novel DNA methyltransferase-1 (DNMT1) depleting anticancer nucleosides, 4'-thio-2'-deoxycytidine and 5-aza-4'-thio-2'-deoxycytidine. *Cancer Chemother Pharmacol* **74**, 291-302 (2014).
19. Parker, W.B. & Thottassery, J.V. 5-Aza-4'-thio-2'-deoxycytidine, a New Orally Bioavailable Nontoxic "Best-in-Class": DNA Methyltransferase 1-Depleting Agent in Clinical Development. *J Pharmacol Exp Ther* **379**, 211-222 (2021).
20. Swerdlow, S.H. *et al.* The 2016 revision of the World Health Organization classification of lymphoid neoplasms. *Blood* **127**, 2375-90 (2016).
21. Alexandrov, L.B. *et al.* Signatures of mutational processes in human cancer. *Nature* **500**, 415-21 (2013).
22. Helleday, T., Eshtad, S. & Nik-Zainal, S. Mechanisms underlying mutational signatures in human cancers. *Nat Rev Genet* **15**, 585-98 (2014).
23. Nik-Zainal, S. *et al.* Mutational processes molding the genomes of 21 breast cancers. *Cell* **149**, 979-93 (2012).
24. Nik-Zainal, S. *et al.* The genome as a record of environmental exposure. *Mutagenesis* **30**, 763-770 (2015).
25. Alexandrov, L.B. *et al.* The repertoire of mutational signatures in human cancer. *Nature* **578**, 94-101 (2020).
26. Goldberg, L. *et al.* Mutant Idh2 Cooperates with a NUP98-HOXD13 Fusion to Induce Early Immature Thymocyte Precursor ALL. *Cancer Res* **81**, 5033-5046 (2021).
27. Du, W. *et al.* A non-myeloablative conditioning approach for long-term engraftment of human and mouse hematopoietic stem cells. *Leukemia* **32**, 2041-2046 (2018).

28. Liu, W. *et al.* Rational identification of a Cdc42 inhibitor presents a new regimen for long-term hematopoietic stem cell mobilization. *Leukemia* **33**, 749-761 (2019).
29. Trowbridge, J.J. & Starczynowski, D.T. Innate immune pathways and inflammation in hematopoietic aging, clonal hematopoiesis, and MDS. *J Exp Med* **218**(2021).
30. Teodorescu, P., Pasca, S., Dima, D., Tomuleasa, C. & Ghiaur, G. Targeting the Microenvironment in MDS: The Final Frontier. *Front Pharmacol* **11**, 1044 (2020).
31. Balderman, S.R. *et al.* Targeting of the bone marrow microenvironment improves outcome in a murine model of myelodysplastic syndrome. *Blood* **127**, 616-25 (2016).
32. Anand, L. & Rodriguez Lopez, C.M. ChromoMap: an R package for interactive visualization of multi-omics data and annotation of chromosomes. *BMC Bioinformatics* **23**, 33 (2022).
33. R Development Core Team. R: A language and environment for statistical computing. (R Foundation for Statistical Computing, Vienna, Austria, 2023).
34. Tate, J.G. *et al.* COSMIC: the Catalogue Of Somatic Mutations In Cancer. *Nucleic Acids Research* **47**, D941-D947 (2018).
35. Matheson, E.C. & Hall, A.G. Assessment of mismatch repair function in leukaemic cell lines and blasts from children with acute lymphoblastic leukaemia. *Carcinogenesis* **24**, 31-8 (2003).
36. Mullen, N.J. & Singh, P.K. Nucleotide metabolism: a pan-cancer metabolic dependency. *Nature Reviews Cancer* **23**, 275-294 (2023).
37. Human Protein Atlas. (2023).
38. Uhlen, M. *et al.* Proteomics. Tissue-based map of the human proteome. *Science* **347**, 1260419 (2015).
39. Toy, G. *et al.* Requirement for deoxycytidine kinase in T and B lymphocyte development. *Proc Natl Acad Sci U S A* **107**, 5551-6 (2010).
40. Kucab, J.E. *et al.* A Compendium of Mutational Signatures of Environmental Agents. *Cell* **177**, 821-836 e16 (2019).
41. Boot, A. *et al.* In-depth characterization of the cisplatin mutational signature in human cell lines and in esophageal and liver tumors. *Genome Res* **28**, 654-665 (2018).
42. Hoang, M.L. *et al.* Mutational Signature of Aristolochic Acid Exposure as Revealed by Whole-Exome Sequencing. *Science Translational Medicine* **5**, 197ra102-197ra102 (2013).
43. Poon, S.L. *et al.* Genome-Wide Mutational Signatures of Aristolochic Acid and Its Application as a Screening Tool. *Science Translational Medicine* **5**, 197ra101-197ra101 (2013).
44. Ames, B.N., Lee, F.D. & Durston, W.E. An improved bacterial test system for the detection and classification of mutagens and carcinogens. *Proc Natl Acad Sci U S A* **70**, 782-6 (1973).
45. Chu, E.H. & Malling, H.V. Mammalian cell genetics. II. Chemical induction of specific locus mutations in Chinese hamster cells in vitro. *Proceedings of the National Academy of Sciences* **61**, 1306-1312 (1968).

46. Clive, D., Flamm, W.G., Machesko, M.R. & Bernheim, N.J. A mutational assay system using the thymidine kinase locus in mouse lymphoma cells. *Mutation Research/Fundamental and Molecular Mechanisms of Mutagenesis* **16**, 77-87 (1972).
47. Gossen, J.A. *et al.* Efficient rescue of integrated shuttle vectors from transgenic mice: a model for studying mutations in vivo. *Proceedings of the National Academy of Sciences* **86**, 7971-7975 (1989).
48. Jacoby, M.A. *et al.* Subclones dominate at MDS progression following allogeneic hematopoietic cell transplant. *JCI Insight* **3**(2018).
49. Uy, G.L. *et al.* Dynamic changes in the clonal structure of MDS and AML in response to epigenetic therapy. *Leukemia* **31**, 872-881 (2017).
50. Goswami, M. *et al.* Pembrolizumab and decitabine for refractory or relapsed acute myeloid leukemia. *J Immunother Cancer* **10**(2022).
51. Chiappinelli, K.B. *et al.* Inhibiting DNA Methylation Causes an Interferon Response in Cancer via dsRNA Including Endogenous Retroviruses. *Cell* **162**, 974-86 (2015).
52. Chung, Y.J., Choi, C.W., Slape, C., Fry, T. & Aplan, P.D. Transplantation of a myelodysplastic syndrome by a long-term repopulating hematopoietic cell. *Proc Natl Acad Sci U S A* **105**, 14088-93 (2008).
53. Morse, H.C., 3rd *et al.* Bethesda proposals for classification of lymphoid neoplasms in mice. *Blood* **100**, 246-58 (2002).
54. Chervinsky, D.S., Lam, D.H., Zhao, X.F., Melman, M.P. & Aplan, P.D. Development and characterization of T cell leukemia cell lines established from SCL/LMO1 double transgenic mice. *Leukemia* **15**, 141-7 (2001).
55. Yin, M. *et al.* A unique mutator phenotype reveals complementary oncogenic lesions leading to acute leukemia. *JCI Insight* **4**(2019).
56. Matsukawa, T. *et al.* Mcm2 hypomorph leads to acute leukemia or hematopoietic stem cell failure, dependent on genetic context. *FASEB J* **36**, e22430 (2022).
57. Li, H. & Durbin, R. Fast and accurate short read alignment with Burrows-Wheeler transform. *Bioinformatics* **25**, 1754-60 (2009).
58. McKenna, A. *et al.* The Genome Analysis Toolkit: a MapReduce framework for analyzing next-generation DNA sequencing data. *Genome Res* **20**, 1297-303 (2010).
59. Islam, S.M.A. *et al.* Uncovering novel mutational signatures by de novo extraction with SigProfilerExtractor. *Cell Genom* **2**, None (2022).

## Figures

Figure 1

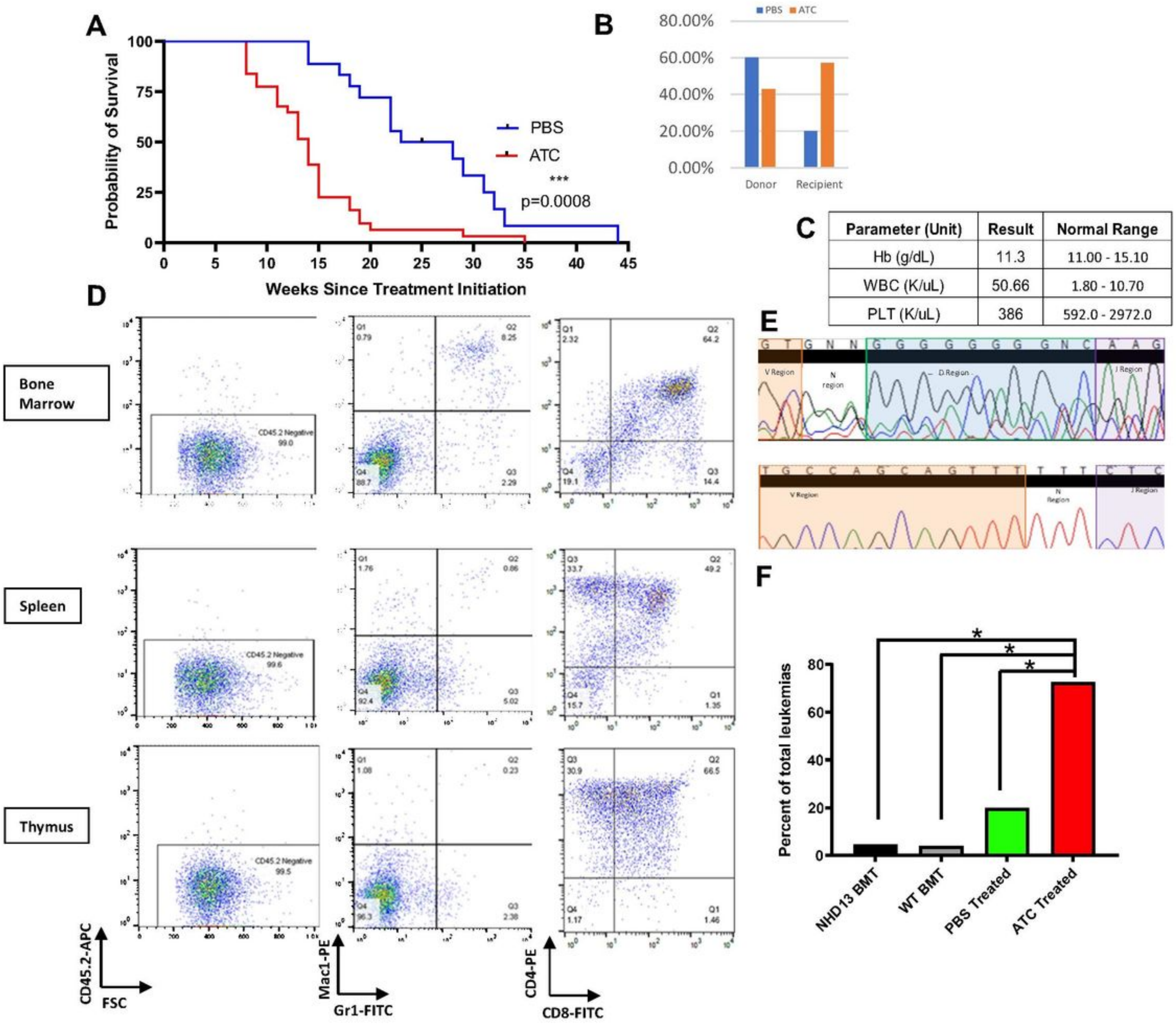

Figure 1

**ATC Treatment leads to T-ALL in recipient cells.**

**A.** Survival curve of pooled results from three independent experiments. **B.** Percent of mice with leukemia of donor (NHD13) vs recipient origin by treatment. **C.** Hematologic parameters of mouse (#3595) with recipient (r)T-ALL. **D.** Flow cytometry dot plot of BM, spleen, and thymus from mouse (#3595) with rT-ALL. **E.** (Top) *Trb* VDJ sequencing of a polyclonal, non-malignant population of thymocytes from a normal thymus. (Bottom) *Trb* VDJ sequencing of a clonal population from mouse 3595 with rT-ALL. **F.** Historical incidence of T-ALL (recipient) in mice from prior NHD13 transplantation experiments utilizing ionizing radiation (IR). Recipient mice were followed for at least 26 weeks. NHD13 BMT refers to mice transplanted with NHD13 BM; WT BMT refers to mice transplanted with WT bone marrow. PBS treated and ATC (0.5-1.0 mg/kg) treated refer to mice in the current study. * indicates p <0.05.

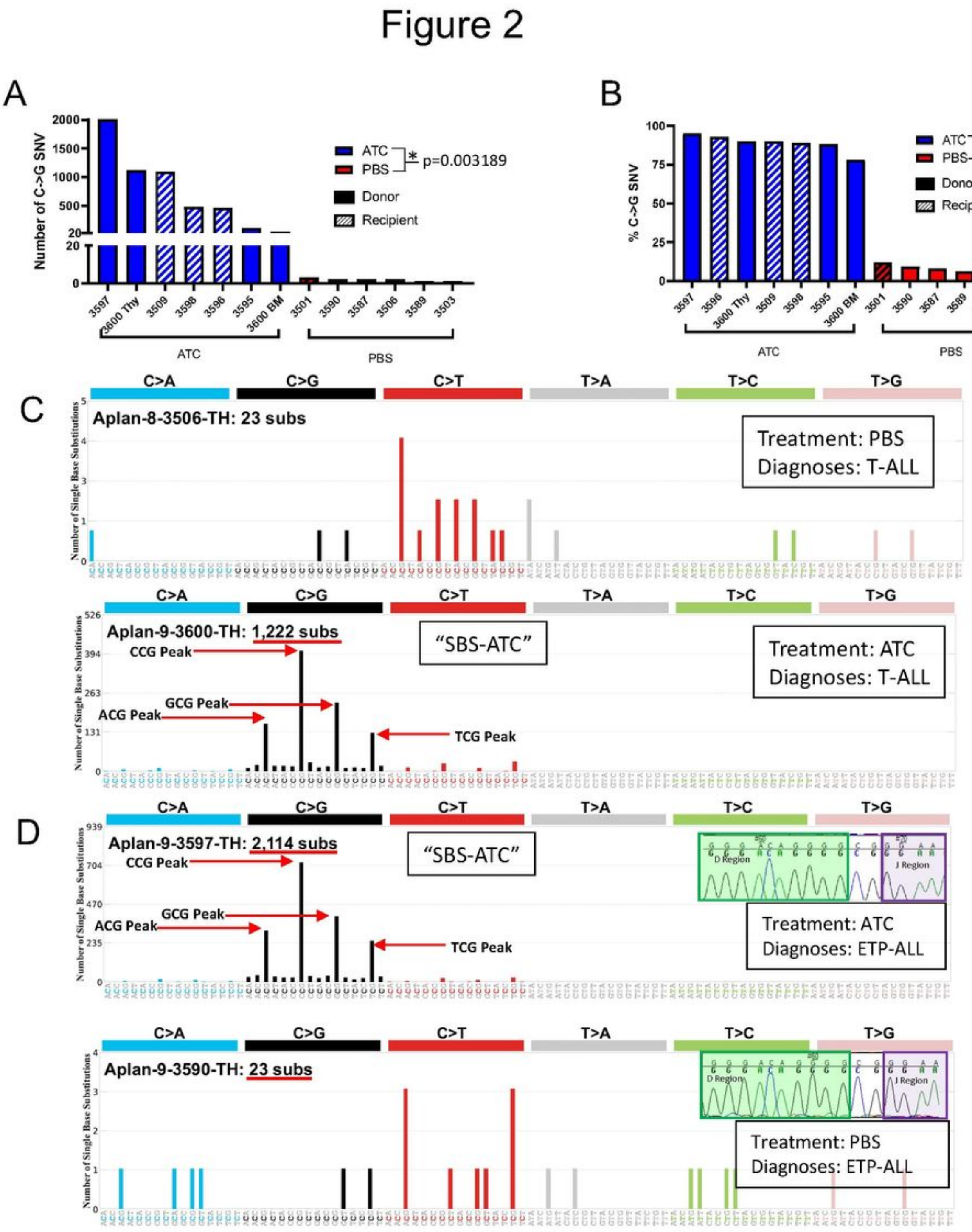

### Figure 2

**Whole Exome Sequencing of ATC-treated mice leukemia reveals a marked increase in C>G Transversions.**

**A.** Absolute number of C>G transversions in leukemias from ATC treated vs. PBS treated mice. **B.** Percent C>G transversions (of all SNVs) in leukemias from ATC treated vs. PBS treated mice. **C.** Single Base Substitution (SBS) plot of mouse with rT-ALL treated with PBS vs. mouse with rT-ALL treated with ATC. Note the marked difference in total number of substitutions and substitutions in a specific 5'-NCG-'3 context ("SBS-ATC"). **D.** SBS plots of mouse with dETP treated with ATC vs. mouse with dETP treated with PBS. The dETP in both of these mice was derived from a pre-leukemic clone transplanted from the NHD13 donor, as documented by identical, clonal *Trb* DJ rearrangements (see inset).

Figure 3

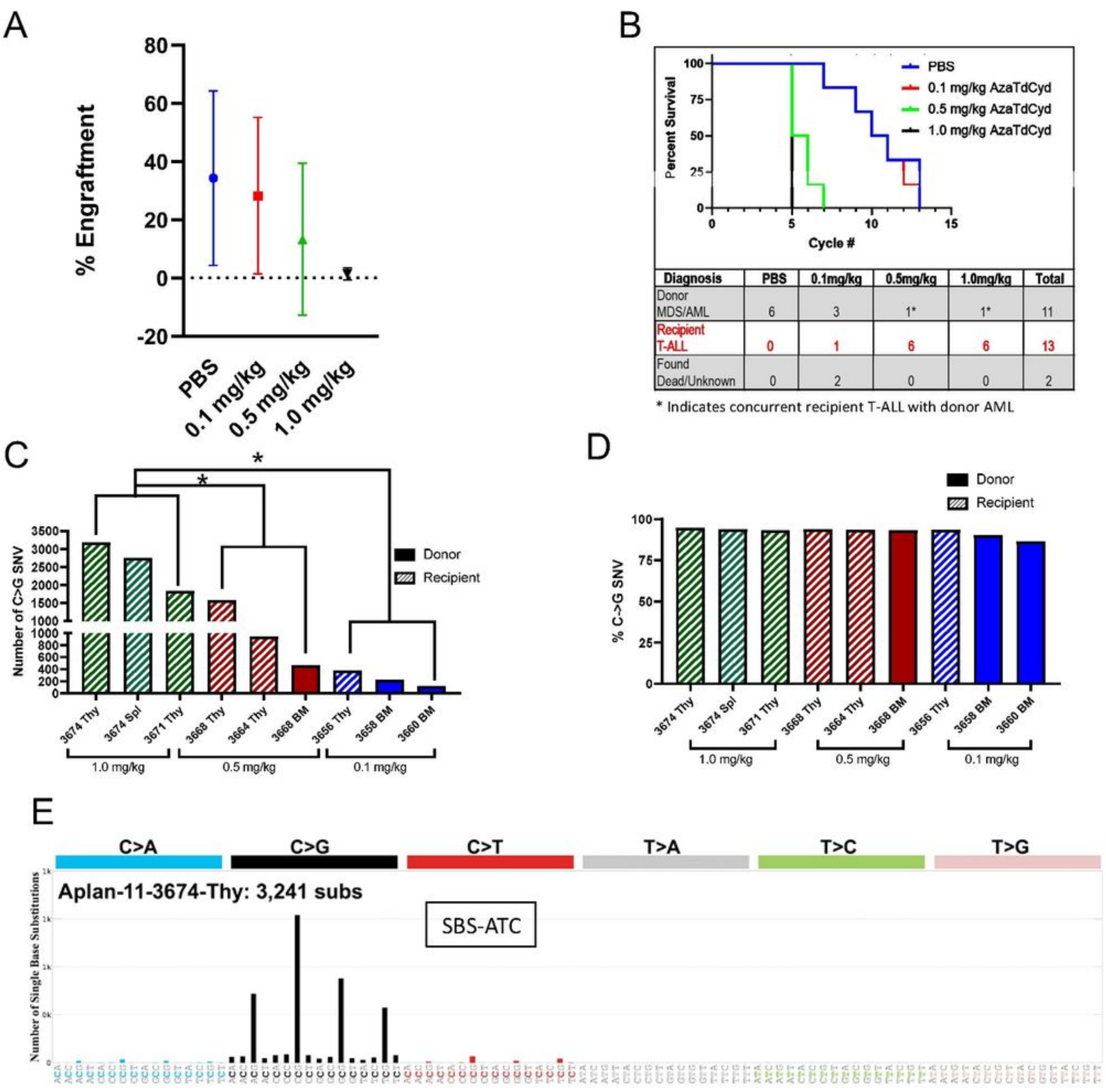

**Figure 3**

**ATC treatment leads to C>G transversion and T-ALL in NHD13 chimeric mice generated without IR.**

**A.** Engraftment of NHD13 cells at time of death in CASIN treated, non-irradiated mice. **B.** Overall survival and cause of death of CASIN conditioned NHD13 chimeric mice by treatment group. **C.** Total number of C>G transversions in treated mice. * indicates p <0.05. **D.** Percent of C>G transversions vs. total mutations **E.** Sanger SBS plot from mouse #3674 treated with ATC demonstrating SBS-ATC.

Figure 4

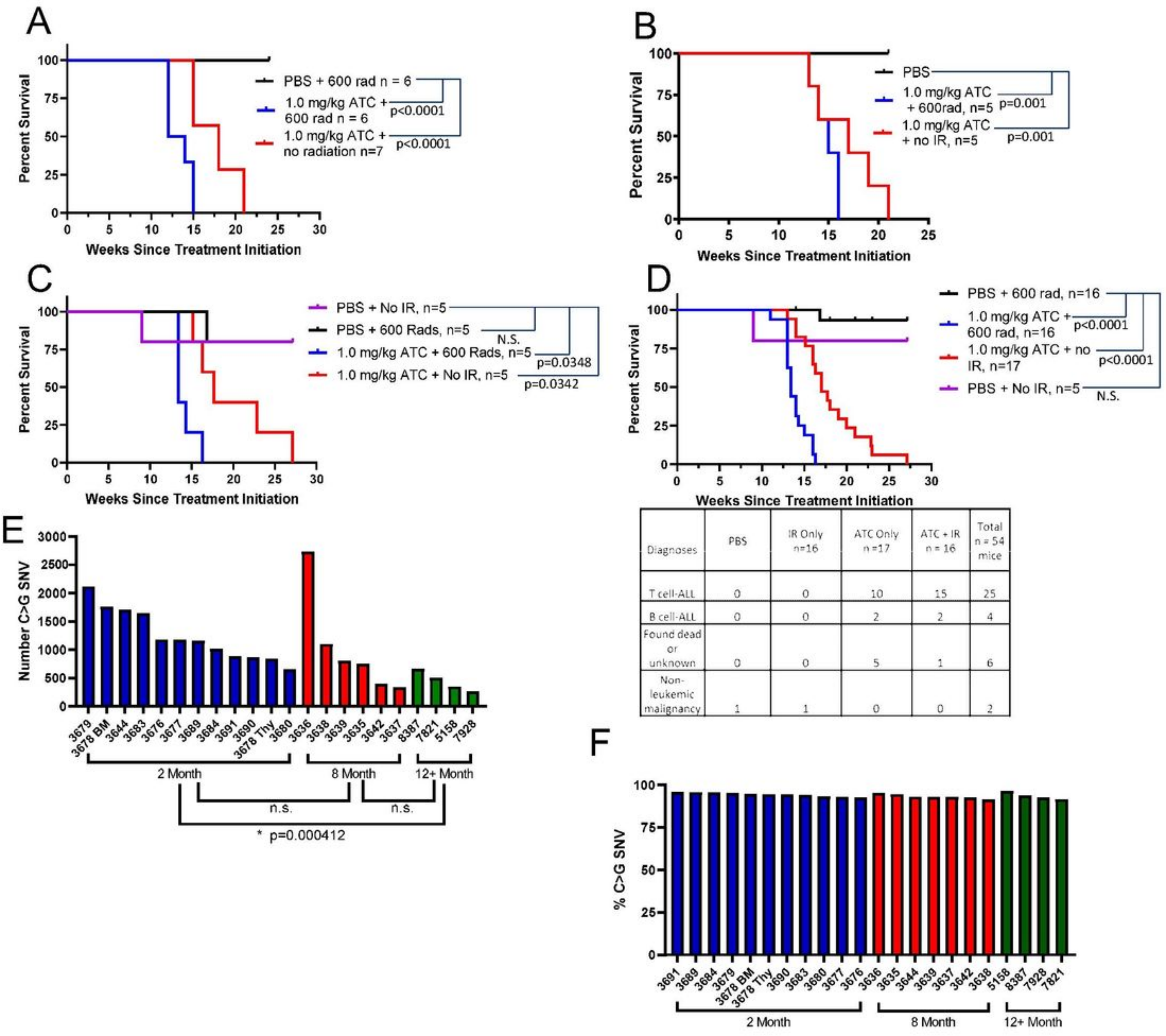

| Diagnoses | PBS | IR Only n=16 | ATC Only n =17 | ATC + IR n = 16 | Total n = 54 mice |
|---|---|---|---|---|---|
| T cell-ALL | 0 | 0 | 10 | 15 | 25 |
| B cell-ALL | 0 | 0 | 2 | 2 | 4 |
| Found dead or unknown | 0 | 0 | 5 | 1 | 6 |
| Non-leukemic malignancy | 1 | 1 | 0 | 0 | 2 |

### Figure 4

#### ATC induces ALL and C>G transversions in a non-transplant setting.

**A.** Survival of 2 month-old C57/BL6 mice treated with ATC +/- IR, **B.** 8 month-old, **C.** 12-15 month-old. **D.** Pooled survival of all 3 experiments and cause of death. **E.** Number of C>G transversions in leukemia of

ATC treated mice. **F.** Percent C>G transversion of all SNV’s in leukemia of ATC treated mice.

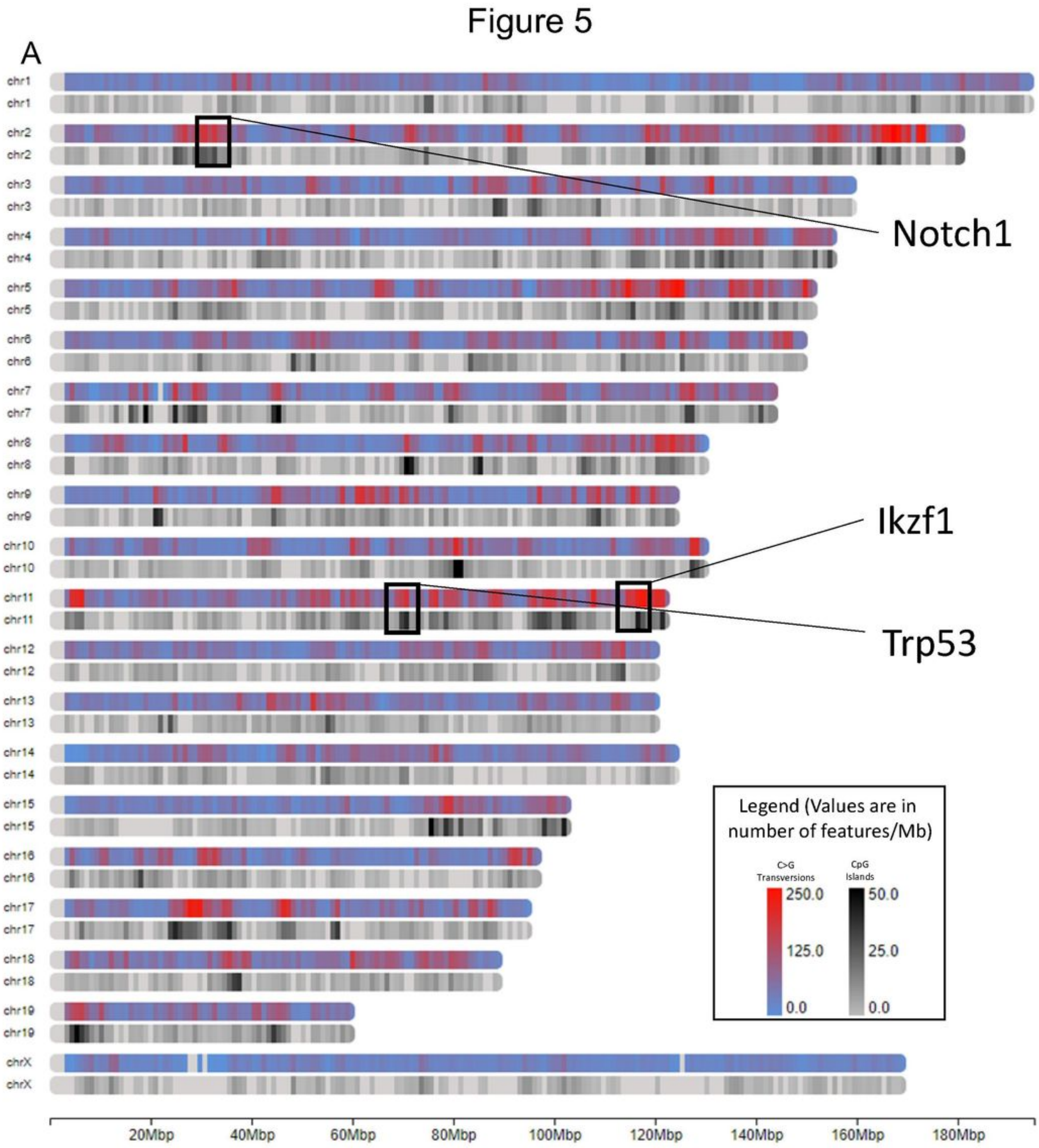

**Figure 5**

**Whole Genome Sequencing reveals C>G transversions across the genome**

**A.** C>G Transversion heat map of 8 sequenced mouse genomes as plotted by chromosome number and region. All mice were treated with ATC. The top chromosome for each pair represents C>G transversion density, whereas the bottom chromosome represents CpG island density.

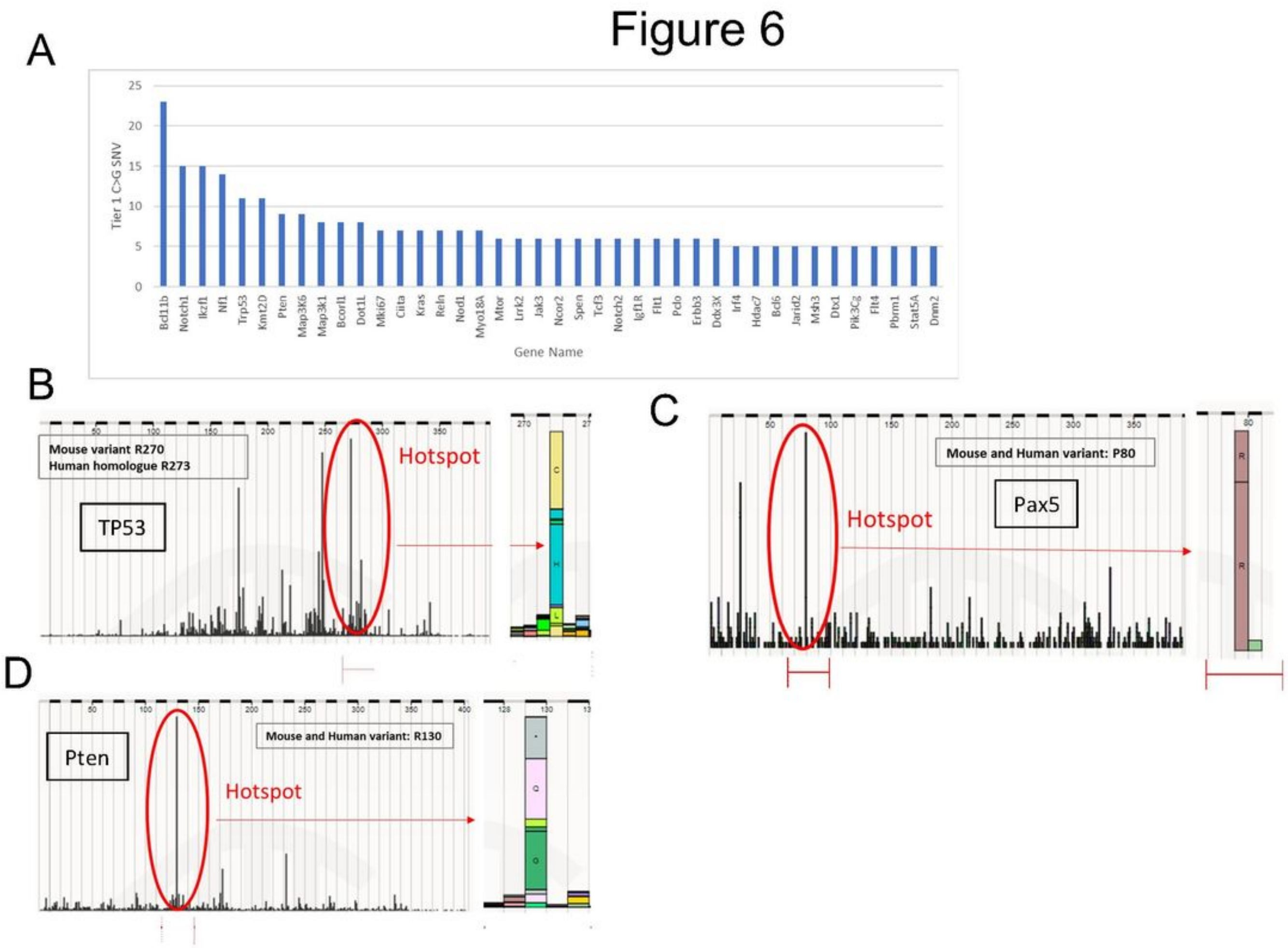

### Figure 6

**Tier 1 SNV induced by ATC occur in genes relevant for human lymphoid leukemia.**

**A.** Tier 1 SNV in genes commonly mutated in human cancer. **B.** Tier 1 mutations induced by ATC are found at residues commonly mutated in human cancer, such as R270P in *Trp53*, **C.** P80R in *Pax5*, and **D.** R130G in *Pten*.

Figure 7

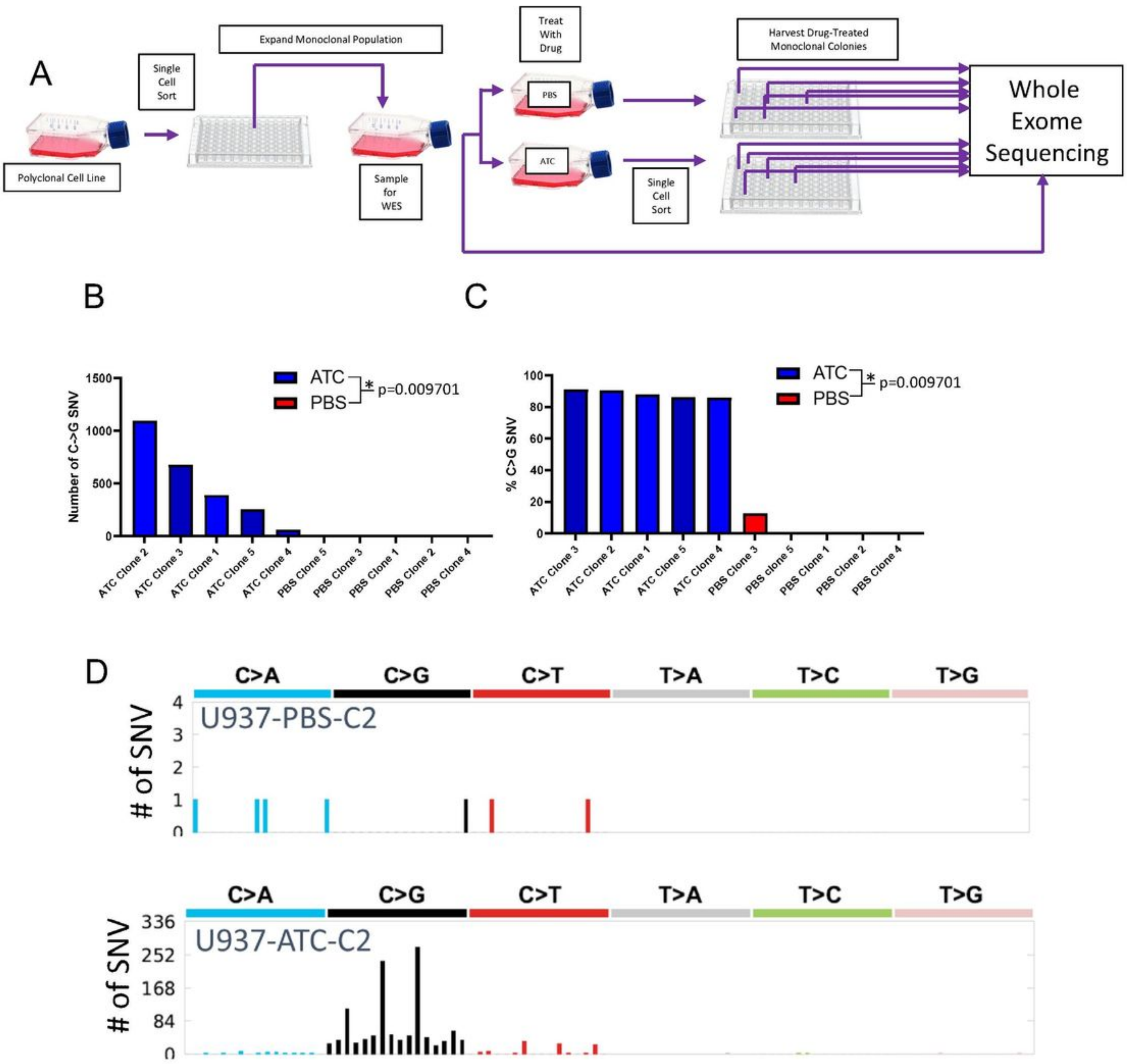

Figure 7

**The human monocytic leukemia cell line U937 develops C>G transversions after treatment with ATC *in vitro.***

**A.** Experimental design of the GEMINI assay used for detecting SNV's induced by drug exposure. **B.** Number of C>G transversion in clonal U937 cell lines treated with either ATC or PBS. **C.** C>G transversions percent of all SNV’s in clonal U937 cell lines treated with either ATC or PBS. **D.** Representative SBS plots of clonal U937 lines treated with ATC vs. PBS. Note that C>G SNV occur preferentially in a 5’-VCG-3’ context.

Figure 8

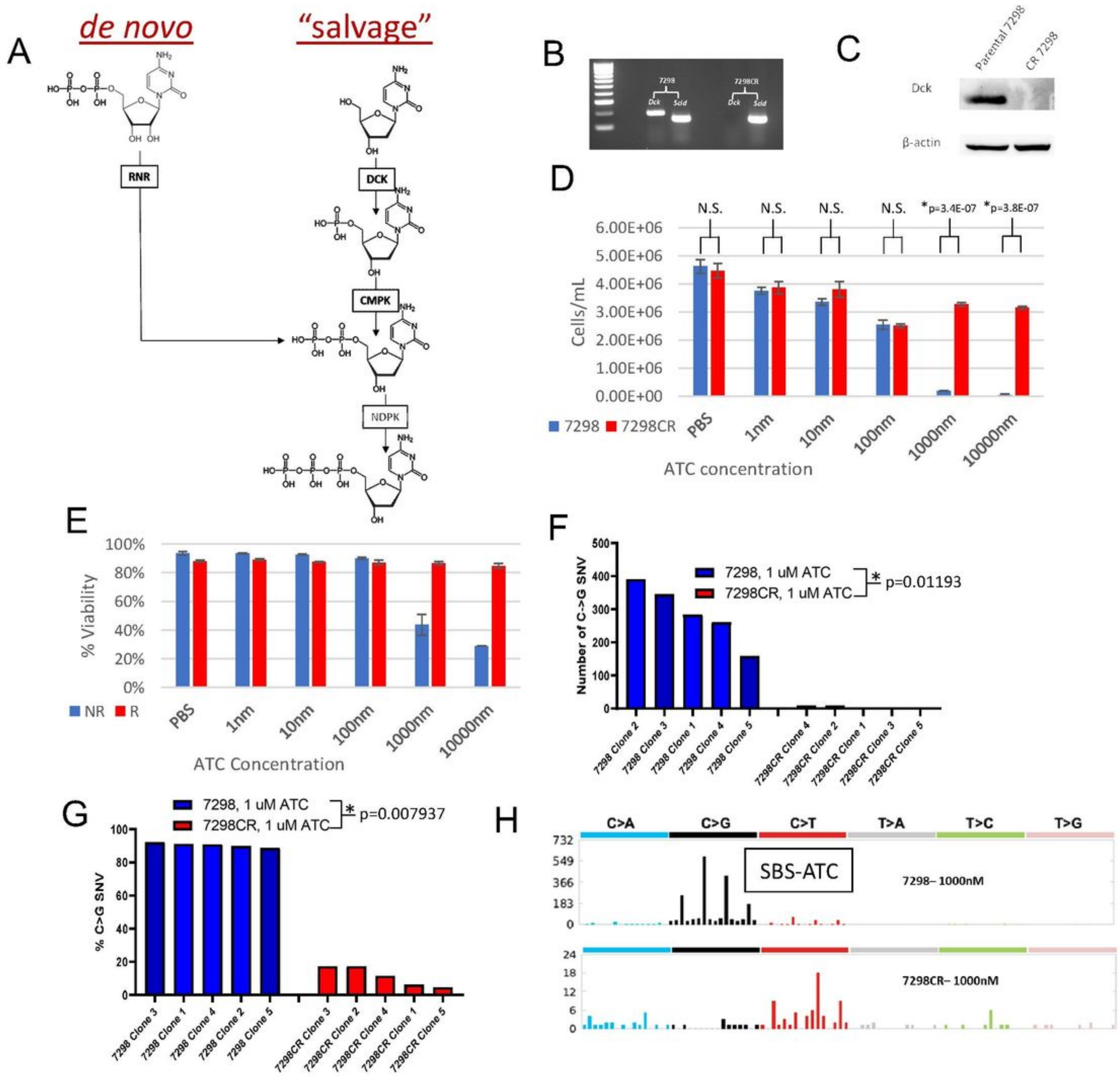

### Figure 8

**A *Dck* deleted cell line is resistant to ATC and does not show increased C>G transversions.**

**A. S**chematic of the pathways available for the generation of a triphosphorylated cytidine or cytidine analog. **B**. Absence of *Dck* intron 6 in 7298CR. Amplification of *Scid* locus used as DNA quality control. **C.** Western blot of Dck in 7298 and 7298CR. **D.** Dck deleted cell line (7298CR; R or resistant) vs. parental Dck competent cell line (7298; NR or non-resistant) cell growth at 3 days in the presence of increasing concentration of ATC. **E.** Percent viability at 3 days. **F.** Absolute number of C>G transversions in 1000 nM ATC treated 7298 vs. 7298CR. **G.** Percent C>G transversions in 1000nM ATC treated 7298 vs. 7298CR. **H.** Representative SBS mutational profiles in a 1000 nM treated 7298 clone and 7298CR clone. Note that 1000 nM 7298 exhibits SBS-ATC whereas 7298CR does not.

## Supplementary Files

This is a list of supplementary files associated with this preprint. Click to download.

- ExtDataTable1TransplantExperimentsCBCandDemographicData.xlsx
- ExtDataTable2TransplantExperimentsFACSandRearrangementData.xlsx
- ExtDataTable3NonTransplantExperimentsCBCandDemographicData.xlsx
- ExtDataTable4NonTransplantExperimentsFACSandRearrangementsData.xlsx
- SuppDataTable1IRandNHD13ExperimentsWES.xlsx
- SuppDataTable2CASINExperimentWES.xlsx
- SuppDataTable3NonTransplantExperimentsWES.xlsx
- SuppDataTable4WholeGenomeSequencing.xlsx
- SuppDataTable5DuplicateCtoGMutationsinWGS.xlsx
- SuppDataTable6MutationsinKnownCancerGenesfromNonTransplantExperiments.xlsx
- SuppDataTable7U937CEMWES.xlsx
- SuppDataTable8DckExperimentsWES.xlsx
- SuppDataTable9PCRPrimers.xlsx
- ExtendedFiguresATCManuscript.pptx
- SupplementalFiguresATCManuscript.pptx
